# Postmortem Abdominal Ultrasound in Healthy Wild Mammals for Application in Forensic Veterinary Medicine

**DOI:** 10.1155/vmi/8878250

**Published:** 2026-02-05

**Authors:** Marlon Ferrari, Sérvio Túlio Jacinto Reis, Naida Cristina Borges, Laila Massad Ribas, Fabiano José Ferreira de Sant’Ana

**Affiliations:** ^1^ Programa de Pós-Graduação em Saúde Animal, Universidade de Brasília (UnB), Brasília, Brazil, unb.br; ^2^ Polícia Federal do Brasil, Instituto Nacional de Criminalística, Brasília, Brazil; ^3^ Escola de Veterinária e Zootecnia, Universidade Federal de Goiás (UFG), Goiânia, Brazil, ufg.br; ^4^ Baroni-Massad, São Paulo, Brazil; ^5^ Laboratório de Diagnóstico Patológico Veterinário, UnB, Brasília, Brazil

## Abstract

Diagnostic imaging methods have been used in human medicine to identify and examine cadavers to determine the cause of death. In veterinary medicine, the use of these resources is still scarce and little known, and it is necessary to establish the contribution that methods such as ultrasound could provide to the investigation of the cause of death in domestic and wild animals. Postmortem ultrasonography (PMUS) can help forensic science find injuries that even in conventional necropsy go unnoticed. Thus, knowing the sensitivity and accuracy of the method is important and, therefore, the present study aimed to evaluate the applicability of the technique and describe the abdominal ultrasound findings in carcasses of wild mammals that died of natural causes and in different states of autolysis. Considering that wild animals are often found in adverse situations, such as the time elapsed before they are discovered, the conservation temperature and environmental conditions that may mask the true state of autolysis, and since ultrasound is portable, this tool becomes important for the forensic expert’s decision‐making. Thus, we hypothesise that the application of PMUS is an important alternative for establishing the thanatological state (TS) and the conditions for performing conventional necropsy. The choice of wild mammals was made due to their similarity with domestic mammals, which facilitates the search for literature and standards. Animals received by the Instituto de Criminalística Nacional (National Forensic Institute) of the Federal Police of Brazil were used. Eighteen wild mammal carcasses were examined, in varying autolytic states: three pumas (*Puma concolor*), three jaguars (*Panthera onca*), two South American coatis (*Nasua nasua*), two giant anteaters (*Myrmecophaga tridactyla*), two white opossums (*Didelphis albiventris*), one pampas deer (*Ozotoceros bezoarticus*), one capybara (*Hydrochoerus hydrochaeris*), one maned wolf (*Chrysocyon brachyurus*), one black‐eared opossum (*Didelphis aurita*), one tayra (*Eira barbara*) and one tapeti (*Sylvilagus brasiliensis*). The accuracy and sensitivity of the ultrasound examination were observed in four TSs: zero 0 (intact), I (mild autolysis), II (moderate autolysis) and III (marked autolysis). The ultrasound evaluation was more sensitive for TS 0 and I; however, it was still possible to evaluate the liver, spleen, kidneys, small intestine and urinary bladder in advanced TS (II and III). The greater the degree of autolysis, the fewer structures and details were visualised. It was concluded that intact or discretely autolysed wild mammals are the most suitable for evaluation by postmortem ultrasound and kidneys and gallbladder were the main structures visualised in cases with advanced autolysis (TS II and III). The findings of this study should be considered preliminary, given that the sampling design was heterogeneous and comprised a limited number of individuals per species.

## 1. Introduction

The search for knowledge about the phenomena of death and illness is not a recent practice, as the first human dissections date back to the 3rd century BC. The introduction of imaging techniques for the evaluation of organs dates back to 1895, with the discovery of X‐rays. Ultrasound imaging forms the image based on the resistance of organs to the passage of sound, and its application in humans and living animals is essential for anatomical studies of tissues rich in water. However, it was only at the beginning of the 21st century that researchers from the Institute of Forensic Medicine at the University of Bern created a project called Virtopsy, which to this day represents the most modern imaging on cadavers [1]. The application of postmortem ultrasound (PMUS) in forensic sciences is still limited, and studies show that many advancements are still needed, considering its practicality, portability and versatility as an imaging technique. This tool can assess the abdominal cavity in a unique and individualised manner, characterising the state of autolysis and the viability of a conventional necropsy.

Ultrasonography is well established in veterinary medicine and routinely used in domestic animals. Moreover, its application proves to be very efficient in live animals, given that the image acquisition method allows the identification of the movement of organs in real time and blood flow in the cardiovascular system [2]. The application of PMUS may provide a method for the evaluation of structures, and it could bring faster identification of focal and diffuse lesions in parenchymal organs, acquisition of biological material through guided collection and differentiation between cadaveric and ante‐mortem changes [3–5]. PMUS can be performed at the location where the carcass was found and assess the thanatological state (TS). Thus, this tool can be used to assess the need for a conventional necropsy with greater or lesser urgency, considering the possibility of autolysis progression.

Through conventional necropsy or PMUS, the TS of the carcass can be classified as O (no autolysis), I (mild autolysis), II (moderate autolysis) and III (marked autolysis). In this technique, the consistency, surface and possible crepitus in the parenchyma of the organs are assessed [6].

In legal medicine, the PMUS technique for forensic purposes is limited to the assessment of tissue impedance, with the possibility of identifying lesions in parenchymal tissues, mainly kidneys, liver and spleen [7, 8]. In forensic veterinary medicine, the basis of the use of PMUS has not yet been described, such as the image acquisition technique, or in which TS is still possible to evaluate the organs. A few previous ultrasound studies have been performed on dog and cat cadavers [4, 9]; however, there are no similar studies on wild mammals. In addition, there are no studies that have investigated how the degree of autolysis specifically impacts the visibility of the organs and tissues of these animals. Considering that wild animals are often found in adverse situations, such as the time elapsed before they are discovered, the conservation temperature and environmental conditions that may mask the true state of autolysis, and since ultrasound is portable, this tool becomes important for the forensic expert’s decision‐making. Thus, the application of PMUS is an important alternative for assessing the TS and the conditions for performing a conventional necropsy.

Therefore, the present study aims to evaluate the applicability of the technique and describe the findings of abdominal ultrasound in cadavers of wild mammals that died naturally and in different states of autolysis. We hypothesise that the degree of autolysis can affect the accuracy and sensitivity of PMUS in some parenchymal organs.

## 2. Materials and Methods

Eighteen wild mammal carcasses were used: three pumas (*Puma concolor*), three jaguars (*Panthera onca*), two South American coatis (*Nasua nasua*), two giant anteaters (*Myrmecophaga tridactyla*), two white‐eared opossums (*Didelphis albiventris*), one pampas deer (*Ozotoceros bezoarticus*), one capybara (*Hydrochoerus hydrochaeris*), one maned wolf (*Chrysocyon brachyurus*), one black‐eared opossum (*Didelphis aurita*), one tayra (*Eira barbara*) and one tapeti (*Sylvilagus brasiliensis*). The carcasses were kindly donated by the Wild Animal Triage Center in Goiás, Brazil, and by the Criminalistics Institute of the Federal Police in Brasília, Distrito Federal. All animals died of natural causes. These animals were used in the current study because they are easily found in the wildlife of Midwestern Brazil, where the institutions involved in this research are located. Because these two public institutions donated all carcasses, our study was deemed exempt from approval by the Institutional Animal Care and Use Committee, according to Brazilian laws (RN 30/2016—DBCA).

Before the ultrasound examination, the animals were thawed at room temperature, the larger ones for 32–48 h and the smaller ones for 8–12 h. Abdominal ultrasound exams were performed by a single operator (MF) between July 2021 and August 2023 using two pieces of equipment. The first, which analysed four animals, was from the brand Mindray Z50 vet., with two transducers: a linear 9–14 Mhz (10L24EA) and a convex 5–9 Mhz (65C15EA).

For the sonographic evaluation, the hair of the abdominal ventral region was cleaned when necessary. Then, a substantial amount of acoustic gel was applied to the animals’ skin to standardise contact between the transducer and skin. The animals were positioned in a supine position to perform sonographic evaluation with the ultrasound equipment. The positioning of the transducer to the achievement of the sagittal and transverse of the urinary bladder, spleen, liver, gallbladder, adrenal, ovary, pancreas and gastrointestinal tract, as well as the evaluation of contour, margin, size, texture and echogenicity of these organs, followed the instructions for domestic pets [2] and wild animals [10, 11].

Next, a necropsy was performed to determine the TS of the animal, as follows: Grade 0—intact, Grade I—mild autolysis, Grade II—moderate autolysis and Grade III—marked autolysis (Table 1) [12].

**TABLE 1:** Gradation of the thanatological states of the wild mammals evaluated in the current study.

Score	Grade	Thanatological states
0	Normal	Fresh or intact after thawing, evidence of rigour mortis, livor mortis and hypostatic congestion
I	Mild autolysis	Mild loss of rigour mortis, moderate hypostatic congestion
II	Moderate autolysis	Marked loss of rigour mortis, friable organs, slight haemoglobin imbibition
III	Marked autolysis	Subcutaneous pseudomelanosis, gaseous distension of the abdomen, emphysema of the organs, detachment of hair, moderate to severe haemoglobin imbibition and liquefaction of the organs

After classifying the carcasses into a TS based on its external features, the ultrasound exam was performed to evaluate the image characteristics and compare them with the images expected in a living animal. In TS 0, the echotexture of the organs corresponded to that expected in a living animal. Thus, it was possible to evaluate each abdominal organ in greater depth and detail. However, as autolysis progressed, tissue integrity deteriorated, leading to alterations in echotexture (TS 1), increased friability associated with intraparenchymal gas, and progressive difficulty in organ evaluation (TS 2). Finally, in TS 3, the organs decrease in size, lose their limits and appear severely friable, with a large amount of intraparenchymal air.

The frequency of organ visualisation by PMUS across different TSs was compared using Fisher’s test with 95% significance (*p* < 0.05). The number of organs analysed per TS is detailed in Tables 2, 3 and 4. Data analysis was performed with STATA software, Version 12 (STATACORP, 2011).

**TABLE 2:** Ultrasonographic findings, absolute (*N*) and percentage (%) values, in carcasses of wild mammals in the thanatological state 0.

Organ	*n* (%)	Ultrasonography findings
Liver	6 (33.3)	The topography was maintained; its attenuation and ultrasound appearance were preserved, hypoechoic, homogeneous, granular and delicate echotexture; the vessels were not visualised; the bile ducts appeared as echogenic structures distributed in a multifocal manner throughout the parenchyma; and the edges were defined
Spleen	6 (33.3)	Hyperechoic, grainy, delicate and homogeneous echotexture, and splenic vessels were not visualised; edges were sharp and defined; and location maintained between the cranial pole of the left kidney and the cardia of the stomach
Kidneys	6 (33.3)	The echotexture was maintained with a 1:1 corticomedullary ratio, hypoechoic cortex and anechoic medulla; the pelvis was characterised by a bright hyperechoic area in the central region of the kidney; and the format was sui generis
Gallbladder	6 (33.3)	Visualised as a cystic structure, elliptical or rounded in shape, with anechoic content, homogeneous or containing little hyperechoic particulate content, a more echogenic wall and well‐defined limits. There was a variation in organ repletion
Urinary bladder	5 (27.7)	It was slightly distended, with generally anechoic and homogeneous content. The wall appeared discreetly irregular with a more pelvic topography
Small intestines	4 (22.2)	Intestinal loops are in atony, with various levels of distension caused by gas, producing reverberation. The parietal thickness and stricture were visualised, as well as the type of content
Adrenal glands	3 (16.6)	Located in the region of the renal hilum, close to the spinal column, bilobed to an elongated shape, with hypoechoic cortex and marked hyperechoic medulla
Stomach	3 (16.6)	The anatomical site was maintained, walls distended, containing reverberation, atony, making it possible to assess the thickness and parietal stricture
Prostate	3 (16.6)	A rounded, hypoechoic, granular and delicate structure was noted, with defined edges, caudal to the urinary vesicle in the topography of the urethra
Pancreas	2 (11.1)	The right pancreatic lobe appeared as a hyperechoic structure adjacent to the wall of the duodenum, with a slightly irregular contour and a granular and delicate echotexture. The pancreatic duct was visualised as a hypoechoic linear structure
Mesenteric lymph nodes	1 (5.5)	Visualised as hypoechoic, circumscribed, elliptical structures adjacent to the intestinal loops
Large intestines	1 (5.5)	It was moderately distended, allowing assessment of the wall thickness and contents of the descending colon and rectum.

*Note: n*: number of animals.

**TABLE 3:** Ultrasonographic findings of absolute (*N*) and percentage (%) values, in carcasses of wild mammals in the thanatological state I.

Organ	*n* (%)	Ultrasonography findings
Kidneys	5 (27.7)	Echotexture and corticomedullary relationship were maintained; however, the cortex was slightly hyperechoic, elongated and exerting minimal pressure with the probe caused shape distortion. The capsule was slightly hyperechoic
Spleen	4 (22.2)	The parenchyma became more hyperechoic and lost edge definition. The gaseous content of the gastrointestinal tract made it difficult to visualise the organ
Liver	4 (22.2)	The echotexture was maintained, but the edges were slightly less defined; the bile ducts were more visible; and the parenchyma remains homogeneous, granular and delicate
Small intestines	3 (16.6)	Segments began to lose striation and the gas content made assessment difficult; however, it was possible to measure and evaluate the content in some regions
Stomach	3 (16.6)	The wall is more distended with gas, generating a lot of reverberation and making it difficult to assess the thickness and parietal striation
Gallbladder	3 (16.6)	It has less repletion; the wall was not well defined, and however, the content remained anechoic. There was some dorsal displacement due to liver changes. Occasionally, visualisation was compromised by reverberation of the stomach and intestinal loops
Urinary bladder	3 (16.6)	The lack of repletion and the beginning of autolysis left the organ retracted in the pelvis, making visualisation difficult. Furthermore, the reverberation of the intestinal loops formed a barrier to the location of the organ
Adrenal glands	1 (5.5)	There was less distinction between the cortex and the medulla
Large intestines	1 (5.5)	The lagomorph animal presented a cecum with a lot of gaseous content, making it possible to evaluate the wall and contents; however, it did not allow the visualisation of the other abdominal structures
Pancreas	1 (5.5)	It is possible to find the topography of the right pancreatic lobe; however, it became hypoechoic

*Note: n*: number of animals.

**TABLE 4:** Ultrasonographic findings, absolute (*N*) and percentage (%) values, in carcasses of wild mammals in thanatological states II and III.

Organ	*n* (%)	TS	Ultrasound findings
Spleen	2 (11.1)	II	Assessment was restricted to the cranial pole, as gas from the small intestine made the visualisation very difficult and, with autolysis, probe manoeuvres were almost inefficient
Liver	4 (22.2)	II	The edges tapered and became discontinuous. The pressure from the probe distorted the organ in some cases. The parenchyma remained hypoechoic, but was no longer homogeneous. The bile ducts were not evident and multifocal hyperechoic granules appeared, suggesting intraparenchymal gas
Small intestines	4 (22.2)	II	The parietal striation lost clarity, and the gas made it difficult to evaluate the segments
Kidneys	4 (22.2)	II	The corticomedullary relation was altered, with the medulla being larger, and the cortex more hyperechoic and thinner. Few hyperechoic granules could be visualised, suggesting intraparenchymal gas. The probe pressure changed the elongated shape of the organ
Gallbladder	4 (22.2)	II	The wall was almost nonexistent, and the shape was generally more elongated. With the reverberation produced by the gastrointestinal tract, visualisation became difficult and the pressure of the probe collapsed the walls, occasionally
Urinary bladder	3 (16.6)	II	The wall became hypoechoic, irregular and agglomerated (empty) in appearance
Spleen	1 (5.5)	III	There was loss of integrity of the organ, suggesting that it was friable, with poorly defined edges, hyperechoic, grainy and delicate echotexture, without clear limits according to probe pressure
Stomach	1 (5.5)	III	The parietal striation disappeared, visualisation was discontinuous, and its limits were poorly defined due to the advancement of autolysis
Liver	2 (11.1)	III	The edges were no longer distinguishable, and the reverberation of the intestinal loops made it difficult to fully characterise the organ. There was a reduction in volume, without visualisation of the hepatic lobes. The hyperechoic granules increased and the organ lost its integrity, suggesting a friable appearance
Small intestines	2 (11.1)	III	There was a loss of the striation, the loops collapsed, and, in some cases, the gas in the small and large intestines formed a barrier; only in a few places, it was possible to clearly see the wall
Large intestines	1 (5.5)	III	The loop became more distended, and it was possible to see the wall and differentiate it from the wall of the small intestine; however, if the distension was accentuated, the quality of visualisation of the other structures of the abdomen was lost
Kidneys	1 (5.5)	III	They became elongated, the cortex thin with a medullary anechoic and heterogeneous echotexture. The hyperechoic granules, multifocal to coalescent, increased in quantity, and the organ became similar to a cystic structure
Urinary bladder	2 (11.1)	III	It was visualised as an agglomerated structure, with a hypoechoic wall, with or without content, or very little distension and loss of its limits

*Note: n*: number of animals.

Abbreviation: TS, thanatological state.

## 3. Results

According to characteristics of the graduation of the TSs confirmed by the conventional necropsy, the TS of the 18 cadavers was as follows: 0 (*n* = 6), I (*n* = 5), II (*n* = 4) and III (*n* = 3). In 17 animals, it was possible to evaluate the abdominal organs; however, as the degree of autolysis increased, fewer structures and details were visualised.

In TSs 0 and I, visual inspection of the carcasses had hair stuck to the skin, a characteristic odour, and little or no apparent secretion. During manipulation, the rigour was still noticeable and the consistency of the skin was firm. During the ultrasound examination, there was little loss of echotexture, and the organs were in their anatomical location, the capsule and edges, and the echogenicity was maintained. It was possible to establish the echogenicity relationship between the spleen, liver and renal cortex, but as autolysis occurred, and with this loss of integrity and tissue adhesion, the organs lost their echographic appearance.

Table 2 shows details regarding ultrasonographic findings in absolute and percentage values of the organs visualised at TS 0.

Table 3 shows the details regarding ultrasonographic findings in absolute and percentage values of the organs visualised at TS I.

In TSs II and III, advanced autolysis was evident, characterised by severe retraction of the eyes, putrid odour, loss of integrity of the skeletal muscles and loss of skin consistency with detachment of hair. Pseudomelanosis and, in one case, marked subcutaneous emphysema were noted before examination. On ultrasound scanning, the organs had poorly defined limits and loss of integrity, depending on the force that was pressed on the probe. In the kidneys, the cortex became more echogenic and the distinction between medulla and pelvis was lost. In the spleen, the edges became barely noticeable. The liver was condensed into a hypoechoic and homogeneous mass, losing its shape. Punctiform, echogenic and bright areas in these organs indicated intraparenchymal gas. Furthermore, liquid fluid in the cavity was a common finding, and some organs, such as the pancreas, adrenal glands and lymph nodes, were not characterised in the ultrasound evaluation. In one animal with severe subcutaneous emphysema, the probe and frequency selection manoeuvres proved to be inefficient, considering that the reverberation was produced by the subcutaneous gas.

Table 4 describes the ultrasound findings, in absolute and percentage values, of animals classified as TS II and TS III, according to organs that were detectable during the examination.

**FIGURE 1:**
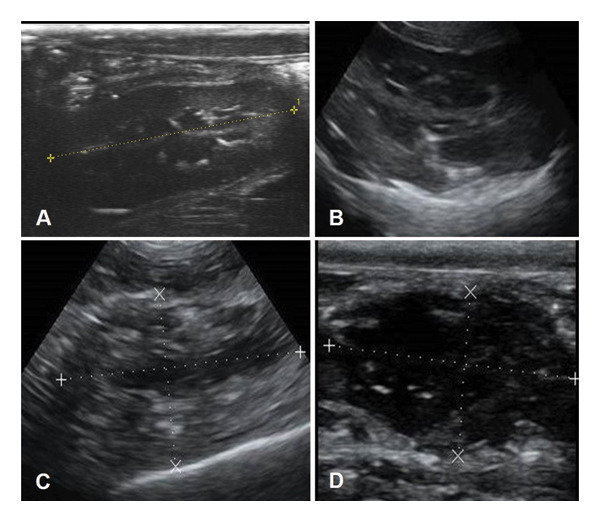
Renal ultrasound in wild animal cadavers in different thanatological states. (A) *Eira barbara*, TS 0. An elongated structure can be seen, with defined edges and a delicate granular echotexture, visualising the three regions (cortex, medullary and pelvis) and a maintained corticomedullary ratio (1:1). The cortex is hypoechoic, the medullary anechoic and the pelvis bright hyperechoic seen as a faint bright line in the central region of the kidney, and the shape is sui generis. (B) *Puma concolor*, TS I. The echotexture and corticomedullary relationship remains maintained; however, the cortex appears a little more hyperechoic, its shape appears elongated, the pelvis is still characterised, and the capsule appears to become a little more hyperechoic. (C) *Chrysocyon brachyurus*, TS II. The corticomedullary relationship is altered, with the medullary gland larger, and the cortex more hyperechoic and thinner, the shape becomes elongated, and hyperechoic granules are visualised in small quantities (intraparenchymal gas). (D) *Puma concolor*, TS III. The cortex becomes thin and appears like a thick capsule, with heterogeneous anechoic content. Hyperechoic granules increase in quantity and the organ becomes similar to a cystic structure, with mixed anechoic content, similar to that seen in abscesses.

**FIGURE 2:**
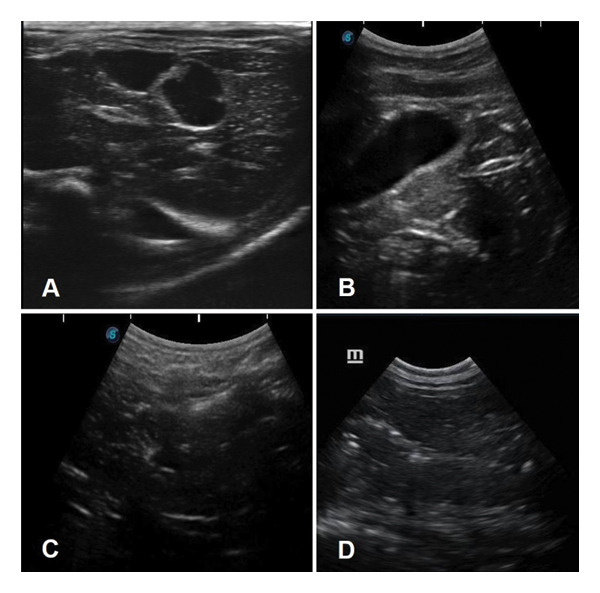
Liver ultrasound performed on wild animal carcasses with different thanatological states. (A) *Nasua nasua*, TS 0. The topography, attenuation and echographic appearance are preserved. A hypoechoic, homogeneous, grainy and delicate echotexture is noted, with evident bile ducts and defined edges. (B) *Panthera onca*, TS I. The echotexture is maintained, but the edges are less defined. (C) *Puma concolor*, TS II. The edges taper and become irregular, the gallbladder is visualised, but the parenchyma is heterogeneous, the bile ducts do not appear, and there are numerous hyperechoic granules (intraparenchymal gas). (D) *Hydrochoerus hydrochaeris*, TS III. The organ becomes less visible, the edges are no longer distinguishable, and the reverberation coming from the intestinal loops makes it difficult to completely characterise the organ. The echotexture becomes more heterogeneous, and there are more hyperechoic granules due to the coldness of the organ and a marked loss of tissue integrity.

**FIGURE 3:**
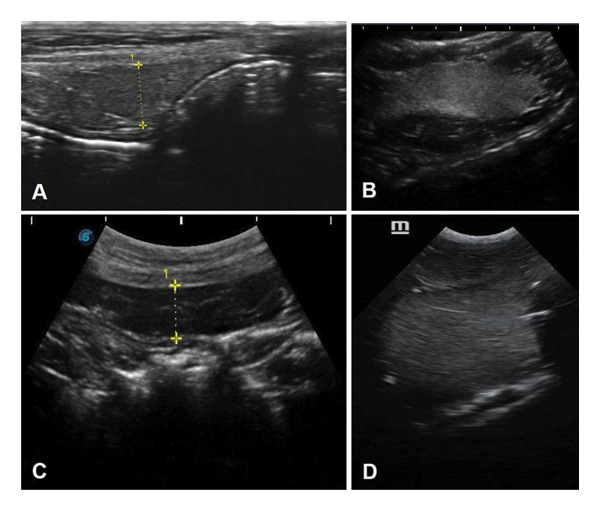
Ultrasound of the spleen of wild animal carcasses with different thanatological states. (A) *Nasua nasua*, TS 0. The echotexture is hyperechoic, grainy, delicate and homogeneous. The vessels are not visualised, the edges are tapered and defined, and the organ is located between the cranial pole of the left kidney and the cardia of the stomach. (B) *Panthera onca*, TS I. The parenchyma is more hyperechoic and edge definition is lost. (C) *Puma concolor*, TS II. The most cranial portion of the organ is visualised and the gas in the intestinal loops makes visualisation very difficult, even with probe manipulation manoeuvres. (D) *Puma concolor*, TS III. There is a loss of integrity of the organ, suggesting that it is friable. The spleen is visualised as a hyperechoic, granular, delicate, homogeneous area, with a macular appearance and poorly defined edges.

Additionally, Figures 1, 2 and 3 illustrate the ultrasound aspects of the kidney (Figure 1), liver (Figure 2) and spleen (Figure 3), respectively.

The autolysed organs became more friable, and, on ultrasound images, they decreased in size with poorly defined edges. The urinary bladder and gallbladder lost the definition of their walls and a reduction in the contents, which made sonographic visualisation difficult. In general, it was observed that as the TSs progressed, it became more difficult to visualise the abdominal organs and their morphological characteristics. Even in cases of advanced autolysis (TS III), the kidneys and the gallbladder were the most easily identifiable structures in all TSs (*p* < 0.05).

**FIGURE 4:**
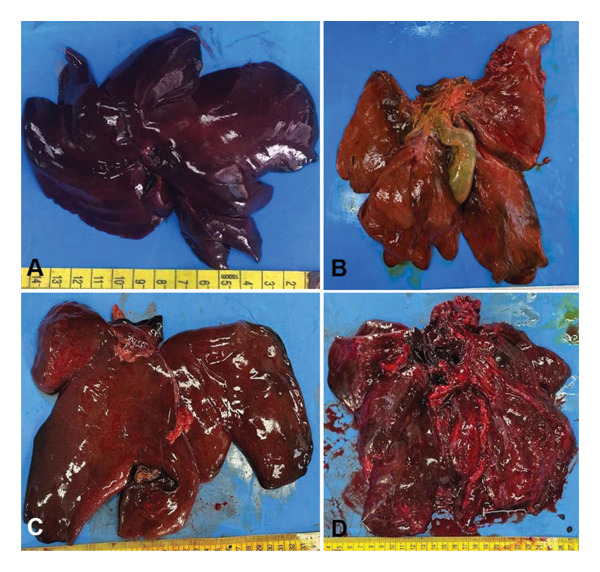
Gross aspect of the liver in thanatological states (TS) 0 to III. (A) TS 0. The appearance and morphology are preserved, with a shiny surface and firm consistency. (B) TS I. There is a loss of consistency, presenting a soft consistency and a moderately full gallbladder. (C) TS II. The consistency is soft to crumbly, and there is a loss of the usual morphology. (D) TS III. The organ is friable, with loss of usual morphology, and the gallbladder is not distended by bile.

The macroscopic appearance of the liver of different wild mammals evaluated in TSs 0 to III is illustrated in Figure 4.

## 4. Discussion

For the first time, an ultrasound study evaluated the abdominal organs of wild mammal carcasses belonging to the Brazilian Cerrado biome, with the aim of identifying the structures and visceral topography, as well as determining whether different states of autolysis interfere with the quality of the evaluation. Thus, the use of PMUS is suggested in forensic practice as a decision‐making tool in validating the time of death and the viability of conventional necropsy, considering the correlation between external examination findings and the evaluation of the morphological integrity of the organs.

In this study, an attempt was made to demonstrate in these species the minimally invasive forensic necropsy technique using PMUS, which is already established in humans [1, 3] and domestic animals [6]. Forensic studies with this scope are relevant in forensic veterinary medicine, considering that wild animals are common victims of environmental crimes and traffic, including in Brazilian territory.

As this study evaluated cadavers with different times of death, some animals presented more advanced TS (II or III). In general, it was observed that the more advanced the degree of autolysis, the lower the accuracy of the technique, making it impossible to identify some structures, especially smaller ones, such as adrenal glands and reproductive organs. These smaller organs are also difficult to assess by ultrasound in live animals [13]. Similar data were observed in previous studies carried out on human cadavers [5]. In the present study, the main effects of autolysis that compromised the morphology and evaluation of the organs included loss of definition of organic limits, loss of volume and formation of intraparenchymal gas. However, the liver, spleen and kidney proved to be viable even in advanced TS. Histologically, the cells of these organs begin to lose definition in autolytic processes, but the general anatomical structure of the organ remains viable for longer. The authors hypothesise that these organs can be more resistant to autolysis due to many factors, such as their structural integrity, rich blood supply and unique cellular features. Other authors also consider autolysis to be an important obstacle to the application of PMUS [5, 6, 14].

In living dogs, the spleen, liver and renal cortex relation is used as a basic characteristic for the interpretation of ultrasound patterns, and, in cases of focal or diffuse lesions, it is the reference for the characterisation of these lesions [15, 16]. Similar conditions are applied in ultrasound studies for standardisation in wild animals [11, 17]. In the present study, these ultrasonographic characteristics were maintained; however, as autolysis progressed, variations occurred in the echotexture and structure of the organs, such as loss of definition of the organ’s limits, increased attenuation, formation of hyperechoic granules, loss of anatomical structure, thinning of the edges and reduction in parenchymal volume.

Comparatively, the spleen remained hyperechoic; still, as autolysis progressed, it lost the definition of its edges, and in TS III, it lost the visualisation of its anatomical site. The liver, despite being visualised in animals with advanced autolysis, suffered a reduction in volume, with thinner edges; the parenchyma was hypoechoic, with a granular and coarse appearance and with discrete multifocal to coalescent hyperechoic granules, consistent with the loss of the organ’s architecture. Recent studies have demonstrated that advanced autolysis markedly limits the application of ultrasound to cadavers [3, 5, 14], as previously investigated [12, 18].

The main difficulties in visualising organs and structures were noted in animals with TS III, resulting from marked subcutaneous emphysema that was observed, which made visualisation and evaluation of the abdominal organs unfeasible, since the gas created a large reverberation artefact at the interface between the skin and the abdomen. Reverberation is an artefact produced when there is gas at the interface between the tissue and the transducer, forming a barrier between the sound beam and the abdomen, making the ultrasound examination unfeasible [2]. Looking for different approaches is an alternative if there is no subcutaneous emphysema.

The ultrasound examination made it possible to observe that with the progression of autolysis, changes in both echogenicity, organ stiffness, the edges of the viscera and anatomical sites occur. Thus, the TS guides the individual condition of each organ and enables an expert to assess which necropsy procedures and material collection methods will be most appropriate, based on ultrasound and the characterisation of the TS. In general, the progress of TSs was strongly related to the visualisation of changes on ultrasound. The greater the degree of autolysis (TS II or III), the more difficult it was to adequately assess the organs.

In addition to traditional necropsy, other advanced imaging techniques, such as computed tomography (CT) and magnetic resonance imaging (MRI), have been employed as alternative methods of forensic investigation in animals [1, 4]. However, our PMUS results indicate the practicality and reliability of the technique, since the portable ultrasound device can be easily transported in field situations, which is not applicable to CT and MRI. The use of PMUS is a major milestone for forensic veterinary medicine studies, as it allows assessment of morphology and lesions in abdominal structures without the need to perform a necropsy, thus making it possible to assist in conventional necropsy by directing to the findings and additionally guiding collections in focal and diffuse organ lesions. Based on the findings of the current study, we suggest that PMUS can be considered a suitable technique to evaluate carcasses of wild animals. In situations of environmental crimes or poaching, PMUS can help to localise focal lesions and foreign bodies, such as firearms projectiles.

This study has some limitations, including the limited number of animals analysed per species, the broad species diversity and the challenge of examining very small organs. Therefore, more studies are needed on different domestic and wild species to evaluate the reliability of this technique in identifying and the degree of agreement between lesions found in PMUS and in conventional anatomopathological examination.

## 5. Conclusions

It was possible to conclude that the PMUS on wild mammal carcasses is useful in forensic veterinary medicine, especially in recently dead animals or in an initial state of autolysis. The knowledge of ultrasound patterns of TSs is important for assessing the autolytic progression of abdominal organs in the carcass, serving as a guide for the decision to perform a conventional necropsy or only collect biological materials, considering the advanced autolytic state. Furthermore, the kidneys and gallbladder are the organs least significantly affected by TSs. New similar studies with larger samples and more species are necessary to reinforce our findings.

## Author Contributions

Marlon Ferrari, Sérvio Túlio Jacinto Reis and Fabiano José Ferreira de Sant’Ana conceptualised the study; Marlon Ferrari and Sérvio Túlio Jacinto Reis developed methodology; Marlon Ferrari, Naida Cristina Borges, Laila Massad Ribas and Fabiano José Ferreira de Sant’Ana performed formal analysis; Marlon Ferrari, Naida Cristina Borges, Laila Massad Ribas and Fabiano José Ferreira de Sant’Ana investigated the data; Marlon Ferrari and Fabiano José Ferreira de Sant’Ana were responsible for resources; Marlon Ferrari, Sérvio Túlio Jacinto Reis, Laila Massad Ribas and Fabiano José Ferreira de Sant’Ana performed data curation; Marlon Ferrari and Fabiano José Ferreira de Sant’Ana prepared the original draft; Marlon Ferrari, Sérvio Túlio Jacinto Reis, Naida Cristina Borges, Laila Massad Ribas and Fabiano José Ferreira de Sant’Ana reviewed and edited the manuscript; Marlon Ferrari, Sérvio Túlio Jacinto Reis and Fabiano José Ferreira de Sant’Ana supervised the study; Marlon Ferrari, Sérvio Túlio Jacinto Reis and Fabiano José Ferreira de Sant’Ana administrated the project.

## Funding

No funding was received for this manuscript.

## Ethics Statement

This project was conducted according to the guidelines and Brazilian Law No. 11794/2008.

## Conflicts of Interest

The authors declare no conflicts of interest.

## Data Availability

The data used to support the findings of this study are available from the corresponding author upon request.
